# Temperature distribution in driven granular mixtures does not depend on mechanism of energy dissipation

**DOI:** 10.1038/s41598-020-57420-0

**Published:** 2020-01-20

**Authors:** Anna S. Bodrova, Alexander Osinsky, Nikolai V. Brilliantov

**Affiliations:** 1 Moscow Institute of Electronics and Mathematics, National Research University Higher School of Economics, 123458 Moscow, Russia; 2 Humboldt University, Department of Physics, 12489 Berlin, Germany; 3 Faculty of Physics, M. V. Lomonosov Moscow State University, 119991 Moscow, Russia; 4 Skolkovo Institute of Science and Technology, 121205 Moscow, Russia; 5 Department of Mathematics, University of Leicester, Leicester, LE1 7RH United Kingdom

**Keywords:** Applied mathematics, Statistical physics

## Abstract

We study analytically and numerically the distribution of granular temperatures in granular mixtures for different dissipation mechanisms of inelastic inter-particle collisions. Both driven and force-free systems are analyzed. We demonstrate that the simplified model of a constant restitution coefficient fails to predict even qualitatively a granular temperature distribution in a homogeneous cooling state. At the same time we reveal for driven systems a stunning result – the distribution of temperatures in granular mixtures is universal. That is, it does not depend on a particular dissipation mechanism of inter-particles collisions, provided the size distributions of particles is steep enough. The results of the analytic theory are compared with simulation results obtained by the direct simulation Monte Carlo (DSMC). The agreement between the theory and simulations is perfect. The reported results may have important consequences for fundamental science as well as for numerous application, e.g. for the experimental modelling in a lab of natural processes.

## Introduction

Mixtures of granular particles of different size are ubiquitous in nature and technology^1–4^. The examples in nature range from pebbles and sands to dust on the Earth. Many extraterrestrial objects are comprised of granular mixtures: One can mention interstellar dust clouds, protoplanetary discs^5^ and planetary rings. Dense Saturn’s rings demonstrate a tremendous size polydispersity, with the particles size ranging from centimeters up to a few meters^6,7^. Granular dust also covers the surface of the Moon^8^, Mars^9^ and possibly other planets and satellites. Industrial granular materials, besides of pebbles and sands in building industry, are represented by powders in chemical and cosmetic production, as well as salt, sugar and cereals in food industry.

Granular materials demonstrate very rich behavior – depending on the applied load they can be in solid liquid or gaseous phase^2^. If the applied load is small, granular materials resist the external force and keep their shape and volume as solids. With increasing external load they start to flow like fluids. Such unusual properties of granular material stem from the dissipative nature of the inter-particle collisions, which are quantified by the so-called restitution coefficient $\varepsilon$, see e.g.^2,10^:

1 $$\varepsilon =|\frac{({{\bf{v}}{\boldsymbol{^{\prime} }}}_{ki}\cdot {\bf{e}})}{({{\bf{v}}}_{ki}\cdot {\bf{e}})}|\,.$$

Here ${{\bf{v}}{\boldsymbol{^{\prime} }}}_{ki}={{\bf{v}}{\boldsymbol{^{\prime} }}}_{k}-{{\bf{v}}{\boldsymbol{^{\prime} }}}_{i}$ and ${{\bf{v}}}_{ki}={{\bf{v}}}_{k}-{{\bf{v}}}_{i}$ are the relative velocities of particles of masses ${m}_{k}$ and ${m}_{i}$ after and before a collision, and **e** is a unit vector directed along the inter-center vector at the collision instant. The post-collision velocities ${{\bf{v}}{\boldsymbol{^{\prime} }}}_{k}$ and ${{\bf{v}}{\boldsymbol{^{\prime} }}}_{i}$ are related to the pre-collision velocities ${{\bf{v}}}_{k}$ and ${{\bf{v}}}_{i}$ as follows, e.g.^10^:

2 $${{\bf{v}}{\boldsymbol{^{\prime} }}}_{k/i}={{\bf{v}}}_{k/i}\mp \frac{{m}_{{\rm{eff}}}}{{m}_{k/i}}(1+\varepsilon )({{\bf{v}}}_{ki}\cdot {\bf{e}}){\bf{e}}.$$

Here ${m}_{{\rm{eff}}}={m}_{1}{m}_{2}$/$({m}_{1}+{m}_{2})$ is the effective mass of colliding particles. The restitution coefficient $0\le \varepsilon  < 1$ shows that the after-collisional relative velocity is smaller than the pre-collisional one, since the mechanical energy is transformed into the internal degrees of freedom of the particles. Due to a permanent loss of the kinetic energy of particles in the collisions, a steady supply of energy is required to keep the system in liquid or gaseous phase (unless the system is in a force-free state, where it undergoes a homogeneous cooling). The nature of the driving forces that fluidize granular matter may be very different. These may be gravitational forces, as in the case of astrophysical objects (dust clouds, protoplanetary disks and planetary rings) or avalanches in mountains^11^. It may be wind of atmospheric gases, initiating the motion of sand grains, which results in dune formation on the Earth^12^ or trigger dust storms on Mars^9^. The fluidization of granular materials in industry may be caused by the vibration of a container, or by moving parts of a system, like e.g. blades or a piston.

The transport properties of granular fluids crucially depend on the mean kinetic energy of the grains, which is also termed as “granular temperature”. Due to dissipative nature of the inter-particles collisions, the energy equipartition, valid for equilibrium molecular systems, does not hold for granular mixtures, where each species has its own temperature. For a mixture of $i=1,2,\ldots N$ species the granular temperature of *k*-th species is defined as follows

3 $$\frac{3}{2}{n}_{k}{T}_{k}=\int \,d{{\bf{v}}}_{k}{f}_{k}({{\bf{v}}}_{k},t)\frac{{m}_{k}{v}_{k}^{2}}{2}$$

Here ${m}_{k}$ is the mass of the according granular species, **v**_*k*_ - its velocity, $f({{\bf{v}}}_{k},t)$ - the velocity distribution function, which quantifies the number of particles in the system of the kind *k* with the velocity ${{\bf{v}}}_{k}$ at time $t$ and ${n}_{k}$ is the number density of the $k$-th species of the granular fluid, ${n}_{k}=\int \,{f}_{k}({{\bf{v}}}_{k},t)d{{\bf{v}}}_{k}$. In what follows we consider granular fluids with a low density, which are termed as “granular gases”. It is expected that the mixture behaves as a gas, when the total packing fraction of all components does not exceed about 20%.

The violation of the energy equipartition in granular mixtures has been recognized almost two decades ago. It was predicted theoretically, confirmed in computer simulations^10,13–16^ and observed experimentally^17,18^. An impressive natural example of a granular mixture with the broken energy equipartition is Saturn rings, where all granular species demonstrate different temperatures^19–22^. The polydispersity in the rings arises due to coagulation and fragmentation of granular particles^7,23^. Although the effect of broken equipartition is known for a long time, still the physical laws that determine the distribution of granular temperatures in granular mixtures are not known. Such laws should predict the granular temperature for each species as a function of (i) size and mass distribution of granular particles, (ii) of the dissipative mechanism of the particles collisions and (iii) of the driving mechanism, applied to the system to keep it fluidized. Force-free granular mixtures can exist in a gaseous state in the regime of homogeneous cooling; here the temperature distribution should be determined by the items (i) and (ii) above. The temperature distribution in granular mixtures with the simplified model of a constant restitution coefficient, $\varepsilon ={\rm{const}}$, which is equal for all inter-particle collisions, has been addressed in our previous study^24^; some universality of the granular temperature distribution was reported^24^. Although the assumption of a constant restitution coefficient drastically simplifies the analysis and is widely used, see e.g.^25–33^, it contradicts, the experimental observations^6,34,35^, as well as basic mechanical laws^36,37^. The latter indicate that $\varepsilon$ does depend on the impact velocity^35,36,38–40^. This dependence may be obtained by solving the equations of motion for colliding particles with the explicit account for the dissipative forces acting between the grains. The simplest, but still rigorous, first-principle model of inelastic collisions takes into account the viscoelastic properties of particles’ material. This results in the corresponding inter-particle forces^38,41^ and eventually, in the restitution coefficient for viscoelastic particles^36,40,42^:

4 $${\varepsilon }_{ki}=1+\mathop{\sum }\limits_{j=1}^{20}\,{h}_{j}{(A{\kappa }_{ki}^{2/5})}^{j/2}{|({{\bf{v}}}_{ki}\cdot {\bf{e}})|}^{j/10},\,{\kappa }_{ki}=\kappa \frac{i+k}{{i}^{5/6}{k}^{5/6}\sqrt{{i}^{1/3}+{k}^{1/3}}},$$

where ${h}_{k}$ are numerical coefficients, $\kappa$ and $A$ characterize respectively the elastic and dissipative properties of the particles material (see Methods). Viscoelastic model agrees well with the experimental data when the impact velocity is not very large^35,43–45^. If the dissipative mechanism is caused by plastic deformation of particles, one obtains the following expression for the restitution coefficient^46^:

5 $$\varepsilon =a(1-{b}^{2}/6){(1+4\sqrt{3/5}\sqrt{{b}^{-2}-{6}^{-2}})}^{1/4}\,a={(6\sqrt{3}/5)}^{1/2}\,b=\frac{{V}_{{\rm{yield}}}}{({\bf{e}}\cdot {{\bf{v}}}_{ki})},$$

where ${V}_{{\rm{yield}}}$ is the yield velocity. Dissipative mechanism associated with plastic deformation corresponds to rather high impact velocities^44,45^. A phenomenological exponential model for the velocity-dependent restitution coefficient has been also employed for the description of experimental data in ref. ^47^,

6 $$\varepsilon =\exp (-\delta \sqrt{\frac{\rho }{Y}}({\bf{e}}\cdot {{\bf{v}}}_{ki})),$$

where *δ* is a dimensionless parameter, $\rho$ is the density of the particle material and *Y* is the Young modulus.

It is well known that the velocity dependence of the restitution coefficient may drastically change the qualitative behavior of granular systems. For instance, it changes the cooling law in a homogeneous cooling state^36,48^, the velocity distribution function^48,49^, the diffusion of granular particles^50^ and even pattern formation, which becomes a transient process for the case of velocity-dependent $\varepsilon$^51^. Therefore, to formulate the laws for the temperature distribution in a granular mixture one needs to consider in detail the dissipation mechanism of particles collision. This is done in the present study. We analyze the distribution of granular temperatures in mixtures of granular gases for different dissipative mechanisms and driving models. We show that the simplified model of a constant restitution coefficient fails to predict even qualitatively the granular temperature distribution in a homogeneous cooling state. At the same time for driven granular systems we arrive at an astonishing result – the distribution of temperatures in granular mixtures is universal, that is, it does not depend on a particular dissipation mechanism of particles collisions. This conclusion holds true for steep distributions of particles size. The results of the analytic theory of the present study are compared with simulation results obtained by the direct simulation Monte Carlo (DSMC). The agreement between the theory and simulations is perfect.

## Results and Discussion

### Model

We consider a polydisperse granular system with discrete distribution of masses of particles. Let the smallest particle mass be ${m}_{1}=0.01$ and masses of other particles read, ${m}_{k}=k{m}_{1}$, where $k=1,2,\ldots N$ are integers and $N$ is the total number of different species in the system. The system is spatially uniform and dilute enough so that only pairwise collisions take place in the system and multiple collisions of the particles may be safely neglected. The mass-velocity distribution function ${f}_{k}({{\bf{v}}}_{k},t)$ gives concentrations of particles of mass ${m}_{k}$ with the velocity ${{\bf{v}}}_{k}$ at time $t$. Since the deviation of the velocity distribution function from the Maxwellian distribution is relatively small^10^, we assume for simplicity that ${f}_{k}({{\bf{v}}}_{k},t)$ is Maxwellian. This function evolves according to the Boltzmann equation, applicable for granular gases, where correlations of velocities of colliding particles may be neglected^10^,

7 $$\frac{\partial }{\partial t}{f}_{k}({{\bf{v}}}_{k},t)=\mathop{\sum }\limits_{i=1}^{N}\,{I}_{ki}^{{\rm{coll}}}+{I}_{k}^{{\rm{heat}}}.$$

In Eq. (7) ${I}_{k}^{{\rm{coll}}}$ is the Boltzmann collision integral^10^:

8 $$\begin{array}{rcl}{I}_{ki}^{{\rm{coll}}} & = & {\sigma }_{ki}^{2}\,\int \,d{{\bf{v}}}_{i}\,\int \,d{\bf{e}}\,\Theta (\,-\,{{\bf{v}}}_{ki}\cdot {\bf{e}})|{{\bf{v}}}_{ki}\cdot {\bf{e}}|\\  &  & \times \,[\chi {f}_{k}({{\bf{v}}{\boldsymbol{^{\prime\prime} }}}_{k},t){f}_{i}({{\bf{v}}{\boldsymbol{^{\prime\prime} }}}_{i},t)-{f}_{k}({{\bf{v}}}_{k},t){f}_{i}({{\bf{v}}}_{i},t)],\end{array}$$

where ${\sigma }_{ki}=({\sigma }_{k}+{\sigma }_{i})/2$, with ${\sigma }_{k}={(6{m}_{k}/(\pi \rho ))}^{1/3}$ being the diameter of particles of mass ${m}_{k}$, $\rho$ is the mass density of the particle material. The summation is performed over all species in the system. ${{\bf{v}}{\boldsymbol{^{\prime\prime} }}}_{k}$ and ${{\bf{v}}{\boldsymbol{^{\prime\prime} }}}_{i}$ are pre-collision velocities in the so-called inverse collision, resulting in the post-collision velocities ${{\bf{v}}}_{k}$ and ${{\bf{v}}}_{i}$. The Heaviside function $\Theta (\,-\,{{\bf{v}}}_{ki}\cdot {\bf{e}})$ selects the approaching particles and the factor $\chi$ equals the product of the Jacobian of the transformation $({{\bf{v}}{\boldsymbol{^{\prime\prime} }}}_{k},{{\bf{v}}{\boldsymbol{^{\prime\prime} }}}_{i})\to ({{\bf{v}}}_{k},{{\bf{v}}}_{i})$ and the ratio of the lengths of the collision cylinders of the inverse and the direct collisions^10^:

9 $$\chi =\frac{|{{\bf{v}}{\boldsymbol{^{\prime\prime} }}}_{ki}\cdot {\bf{e}}|}{|{{\bf{v}}}_{ki}\cdot {\bf{e}}|}\frac{{\mathscr{D}}({{\bf{v}}{\boldsymbol{^{\prime\prime} }}}_{k},{{\bf{v}}{\boldsymbol{^{\prime\prime} }}}_{i})}{{\mathscr{D}}({{\bf{v}}}_{k},{{\bf{v}}}_{i})}$$

In the case of a constant restitution coefficient $\chi =1/{\varepsilon }^{2}$ for viscoelastic particles it has a more complicated form^10^.

The second term ${I}_{k}^{{\rm{heat}}}$ describes the driving of the system. It quantifies the energy injection into a granular gas to compensate its losses in dissipative collisions; it is zero for a gas in a homogeneous cooling state (HCS). Here we consider a uniform heating – the case when the grains suffer small random uncorrelated kicks throughout the volume^52,53^. To mimic the external driving forces a few types of thermostat have been proposed^53^. For a thermostat with a Gaussian white noise, the heating term has the form^53,54^:

10 $${I}_{k}^{{\rm{heat}}}=\frac{1}{2}\frac{{\Gamma }_{k}}{{m}_{k}}\frac{{\partial }^{2}}{\partial {{\bf{v}}}_{{\bf{k}}}^{{\bf{2}}}}{f}_{k}({{\bf{v}}}_{{\bf{k}}},t).$$

Here the constant ${\Gamma }_{k}$ characterizes the strength of the driving force. It may vary for different species, depending on the type of driving^55^. When all species are supplied with the same energy, we have a driving equipartition, ${\Gamma }_{k}={\Gamma }_{1}={\rm{const}}.$ In the case of the force controlled driving ${\Gamma }_{k}\propto 1/{m}_{k}$, while in the case of the velocity controlled driving ${\Gamma }_{k}\propto {m}_{k}$^55^. In our study we analyze a more general case of a power-law dependence of ${\Gamma }_{k}$ on a particle mass, namely, ${\Gamma }_{k}={\Gamma }_{1}{k}^{\gamma }$. The driving may also depend on the local velocity of granular particles^56^, however we neglect this effect in the present study.

Multiplying the Boltzmann Eq. (7) by ${m}_{k}{v}_{k}^{2}$/2 for $k=1\ldots N$ and performing the integration over ${{\bf{v}}}_{k}$, we get the following system of equations for evolution of the granular temperatures of species of different masses:

11 $$\begin{array}{rcl}\frac{d{T}_{1}}{dt} & = & -{T}_{1}\,\mathop{\sum }\limits_{i=1}^{N}\,{\xi }_{1i}+{\Gamma }_{k}\\  &  & \,\ldots \\ \frac{d{T}_{k}}{dt} & = & -{T}_{k}\,\mathop{\sum }\limits_{i=1}^{N}\,{\xi }_{ki}+{\Gamma }_{k}\\  &  & \,\ldots \\ \frac{d{T}_{N}}{dt} & = & -{T}_{N}\,\mathop{\sum }\limits_{i=1}^{N}\,{\xi }_{Ni}+{\Gamma }_{k}.\end{array}$$

In a homogeneous cooling state ${\Gamma }_{k}=0$ and the granular system permanently cools down. Driven granular systems, that is, systems with a thermostat rapidly settle into a non-equilibrium steady state and all granular temperatures attain after a short time, some constant values, so that $d{T}_{k}/dt=0$ and the above system (11) turns into a set of algebraic equations,

12 $${T}_{k}\,\mathop{\sum }\limits_{i=1}^{N}\,{\xi }_{ki}={\Gamma }_{1}{k}^{\gamma }.$$

For the constant restitution coefficient the cooling rate ${\xi }_{ki}$, quantifying the decrease of granular temperature of species of mass ${m}_{k}$ due to collisions with species of mass ${m}_{i}$ is given by the following expression^24^:

13 $$\begin{array}{rcl}{\xi }_{ki}(t) & = & \frac{8}{3}\sqrt{2\pi }{n}_{i}{\sigma }_{ki}^{2}{g}_{2}({\sigma }_{ki}){(\frac{{T}_{k}{m}_{i}+{T}_{i}{m}_{k}}{{m}_{i}{m}_{k}})}^{1/2}\\  &  & \times \,(1+{\varepsilon }_{ki})(\frac{{m}_{i}}{{m}_{i}+{m}_{k}})[1-\frac{1}{2}(1+{\varepsilon }_{ki})\frac{{T}_{i}{m}_{k}+{T}_{k}{m}_{i}}{{T}_{k}({m}_{i}+{m}_{k})}].\end{array}$$

In ref. ^24^ it was assumed that ${\varepsilon }_{ki}=\varepsilon$. In the case of viscoelastic particles the cooling rates may be also computed and the result reads (see Methods for detail):

14 $$\begin{array}{rcl}{\xi }_{ki}(t) & = & \frac{16}{3}\sqrt{2\pi }{n}_{i}{\sigma }_{ki}^{2}{g}_{2}({\sigma }_{ik}){(\frac{{T}_{k}{m}_{i}+{T}_{i}{m}_{k}}{{m}_{i}{m}_{k}})}^{1/2}(\frac{{m}_{i}}{{m}_{i}+{m}_{k}})\\  &  & \times \,[1-\frac{{T}_{k}{m}_{i}+{T}_{i}{m}_{k}}{{T}_{k}({m}_{i}+{m}_{k})}+\mathop{\sum }\limits_{n=2}^{\infty }\,{B}_{n}({h}_{n}-\frac{1}{2}\frac{{T}_{k}{m}_{i}+{T}_{i}{m}_{k}}{{T}_{k}({m}_{i}+{m}_{k})}{A}_{n})]\end{array}$$

where ${A}_{n}=4{h}_{n}+{\sum }_{j+k=n}\,{h}_{j}{h}_{k}$ are pure numbers and

15 $${B}_{n}(t)={(A{\kappa }_{ki}^{2/5})}^{\frac{n}{2}}{(2\frac{{T}_{k}{m}_{i}+{T}_{i}{m}_{k}}{{m}_{i}{m}_{k}})}^{\frac{n}{20}}(\frac{(20+n)n}{800})\Gamma (\frac{n}{20})$$

with $\Gamma (x)$ being the Gamma-function.

While it is possible to obtain the explicit expressions for the cooling coefficients ${\xi }_{ki}$ for the viscoelastic dissipative model, it is not the case for the elasto-plastic model. Therefore it is practical to exploit a notion of a “quasi-constant” restitution coefficient, which corresponds to the effective restitution coefficient averaged over all collisions; it depends on the current temperature of granular fluid^49^, but not on the impact velocity.

### Effective restitution coefficient of colliding particles in a granular mixture

The Eqs. (4), (5) and (6) for the restitution coefficient $\varepsilon$ give this quantity for a collision with a particular impact velocity. Since a wide range of the impact velocities is observed, one deals with a wide range of restitution coefficients. Naturally, this is much more complicated than to deal with a single number, of a simplified model of a constant restitution coefficient. Therefore for practical reasons it is worth to define a restitution coefficient $\langle {\varepsilon }_{ki}\rangle$, averaged over all possible collisions. Such effective “quasi-constant” restitution coefficient may be defined as follows^49^,

16 $$\langle {\varepsilon }_{ki}\rangle =\frac{\int \,d{{\bf{v}}}_{k}d{{\bf{v}}}_{i}d{\bf{e}}f({{\bf{v}}}_{k},t)f({{\bf{v}}}_{i},t)\Theta (\,-\,{v}_{n})|{v}_{n}|{\varepsilon }_{ki}({v}_{n})}{\langle |{v}_{n}|\rangle }.$$

Here ${v}_{n}=({{\bf{v}}}_{ki}\cdot {\bf{e}})$ is the normal component of the relative velocity of particles and $\langle |{v}_{n}|\rangle$ is its collisional average:

17 $$\langle |{v}_{n}|\rangle =2\sqrt{2\pi }\sqrt{\frac{{T}_{k}}{{m}_{k}}+\frac{{T}_{i}}{{m}_{i}}}$$

The Heaviside function $\Theta (-{v}_{n})$ selects the approaching particles. This quantity has been introduced and tested in^49^ for a uniform one-component granular gas and demonstrated its adequacy. Here we generalize this concept for a mixture of granular particles of different masses ${m}_{i}$ and granular temperatures ${T}_{i}$. Using again the Maxwellian approximation for the velocity distribution function, the collisional average yields for the effective coefficient of the viscoelastic particles (see Methods for detail):

18 $$\langle {\varepsilon }_{ki}\rangle =1+\mathop{\sum }\limits_{n=1}^{20}\,\frac{{2}^{1+\frac{n}{20}}}{2+\frac{n}{10}}\Gamma (2+\frac{n}{20}){h}_{n}{(A{\kappa }_{ki}^{2/5})}^{n/2}{(\frac{{T}_{k}}{{m}_{k}}+\frac{{T}_{i}}{{m}_{i}})}^{n/20}.$$

In contrast to the case of one-component granular gas^49^, the effective restitution coefficient depends on the granular temperatures of both components ${T}_{k}$ and ${T}_{i}$, but not on the impact velocity, since it characterizes the collisions in average. The “quasi-constant” restitution coefficient (18) may be now used in Eq. (13) for the cooling rates in place of the constant restitution coefficients; that is, the following substitute may be used ${\varepsilon }_{ki}\to \langle {\varepsilon }_{ki}\rangle$. Although Eq. (18) refers to viscoelastic particles, one apply the collisional averaging (16) for other dissipation models.

### Homogeneous cooling state: The failure of simplified model of constant *ε*

**Figure 1:**
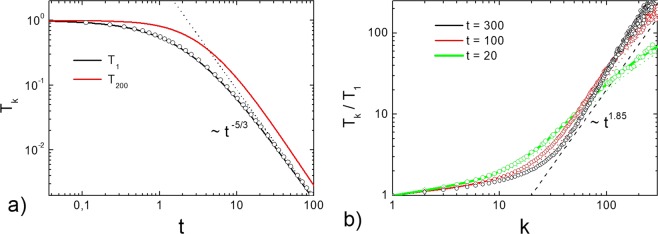
(**a**) Evolution of granular temperatures ${T}_{1}$ and ${T}_{200}$ in the granular gas in a homogeneous cooling statefor the velocity-dependent restitution coefficient, Eq. (4). The dotted line shows the asymptotics $\sim {t}^{-\mathrm{5/3}}$. Symbols show the results of the DSMC simulations. (**b**) Dependence of the granular temperatures ${T}_{k}$ in the HCS on the reduced mass of the particle $k={m}_{k}/{m}_{1}$ at different times. Solid lines correspond to viscoelastic particles (solution of system of Eq. (11) with ${\xi }_{ik}$ in the form Eq. (14) with $A{\kappa }^{2/5}=0.441$. Symbols show the results of the DSMC simulations. The dotted line shows the slope of the temperature distribution for the case of a constant $\varepsilon$.

**Figure 2:**
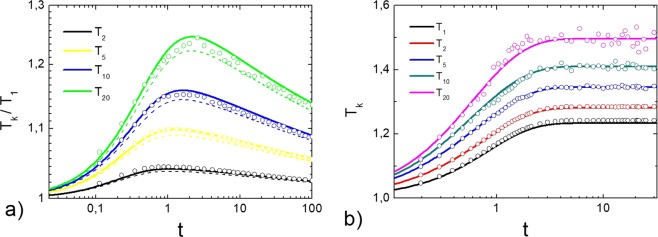
Evolution of granular temperatures ${T}_{k}$. (**a**) In a homogeneous cooling state. Solid lines illustrate the direct solution of the system of Eq. (11) with ${\xi }_{ki}$ in the form of (14). Dashed lines show the evolution of ${T}_{k}/{T}_{1}$ of particles, colliding with an effective restitution coefficient (18) with $A{\kappa }^{2/5}=0.063$. (**b**) In a uniformly heated granular gas with ${\Gamma }_{k}={\Gamma }_{1}=1$ (all species are supplied with the same energy) for $A{\kappa }^{2/5}=0.063$. The notations are the same as in the panel (a).

In order to calculate the granular temperatures of particles of different masses in the homogeneous cooling state we have solved numerically the system of differential Eq. (11) with zero heating rate, ${\Gamma }_{k}=0$ and cooling coefficients ${\xi }_{ki}$ (Eq. 14), corresponding to viscoelastic particles. We exploit the size distributions of particles, which is steep enough, ${n}_{k}\simeq {k}^{-\theta }$ with $\theta =3$. The evolution of granular temperatures ${T}_{k}(t)$ is shown in Fig. 1a. The behavior of a granular gas of viscoelastic particles is drastically different as compared to the granular gas of particles colliding with a constant restitution coefficient. While in the case of constant restitution coefficient the granular temperature decrease with the same rate, corresponding to the Haff’s law^10^, the evolution of granular temperatures of viscoelastic particles is rather complicated. The temperature of monomers ${T}_{1}$ of mass ${m}_{1}$ cools down according to ${t}^{-\mathrm{5/3}}$ (the generalized Haff’s law^10^), while the temperature of more massive particles decrease slower at the initial state of cooling (retarded cooling) and faster at the later state of cooling (accelerated cooling) (Fig. 1a). The difference becomes more pronounced with increasing mass, as it is shown in Fig. 2a, where the evolution of the ratios of granular temperatures is shown. In the case of a constant restitution coefficient the ratios of all granular temperatures tend to the steady state^49^, while for viscoelastic particles the ratio of granular temperatures, ${T}_{k}$/${T}_{1}$, first grows, then reaches its maximal value and then decreases with time, tending to unity, that is, tending to the equipartition (Fig. 2a). The larger the mass ${m}_{i}$ of the particle, the larger the granular temperature and at the later time the maximum of ${T}_{k}$/${T}_{1}$ is achieved. The temperature distribution ${T}_{k}$/${T}_{1}$ evolves with time and changes its form, see Fig. 1b. It does not correspond to the distribution ${T}_{k}\sim {k}^{1.85}$, observed for the system with a constant restitution coefficient^24^. Hence the behavior of granular temperatures with a realistic restitution coefficient qualitatively differs from that predicted for a model with a constant $\varepsilon$. In other words the simplified model of a constant restitution coefficient, widely used in the scientific literature, fails to describe a complicated behavior of a granular mixture. At the same time, as it is follows from Fig. 2a, the application of the corresponding quasi-constant, temperature-dependent restitution coefficient (18) allows to model a granular mixture with an acceptable accuracy. Figures 1 and 2 also demonstrate that the theoretical results obtained by the solution of the rate Eq. (11) are in a perfect agreement with the numerical simulations by the DSMC (see Methods for more detail).

### Driven granular mixtures: Universality of temperature distribution for all dissipative mechanisms

**Figure 3:**
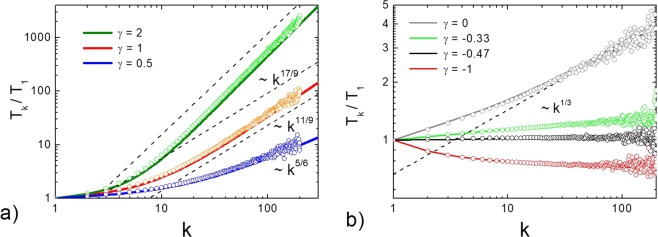
Dependence of the granular temperatures ${T}_{k}$ on the reduced mass of the particle $k={m}_{k}/{m}_{1}$ for a heated granular gas with ${\Gamma }_{k}={k}^{\gamma }$. (**a**) For $\gamma =1$ (velocity controlled driving), $\gamma =2$ and $\gamma =0.5$. The results of the solution of the system with the effective restitution coefficient are shown with thin lines (cooling rates ${\xi }_{ki}$, Eq. (13) with the restitution coefficient $\langle {\varepsilon }_{ki}\rangle$, Eq. (18)), while the full solution of the system is given by the thick lines (cooling rates ${\xi }_{ki}$, Eq. (14)). Two solutions are practically indistinguishable. Symbols show the results of the DSMC simulations. The viscoelastic parameter is $A{\kappa }^{2/5}=0.063$. (**b**) For $\gamma =0$ (equal distribution for the energy input for all species) and negative values of the exponent *γ*: $\gamma =-\,0.33,-\,0.47,-\,1$. The notations are the same as for the panel (a). The viscoelastic parameter is $A{\kappa }^{2/5}=0.441$.

To study the evolution of a driven granular mixture we solve the system of Eq. (11) with the mass-dependent heating rates ${\Gamma }_{k}={\Gamma }_{1}{k}^{\gamma }$. We start from the case of viscoelastic particles and use the cooling coefficients (14) and then the cooling coefficients (13) for the impact-velocity independent restitution coefficients ${\varepsilon }_{ki}$, with the use of the effective quasi-constant coefficients, $\langle {\varepsilon }_{ki}\rangle$ from Eq. (18) in the place of ${\varepsilon }_{ki}$. The evolution of granular temperatures in a heated gas is shown at Fig. 2b. As one can see from the figure, all temperatures relax to the steady-state values. The steady state temperatures ${T}_{k}$ form a stationary distribution, that behaves for large $k$ as a power law, see Fig. 3.

To understand this behavior theoretically, we assume the power-law distribution for the steady state temperatures,

19 $${T}_{k}={T}_{1}{k}^{\alpha },$$

provided *k* are not small. Approximating for $N\gg 1$ the summation by integration in Eq. (12) with the cooling rates, Eq. (14), we get:

20 $$\begin{array}{rcl}\mathop{\sum }\limits_{i=1}^{N}\,{\xi }_{ki} & = & c\,{\int }_{1}^{N}\,di{n}_{i}{({i}^{1/3}+{k}^{1/3})}^{2}\frac{i}{k+i}{({i}^{\alpha -1}+{k}^{\alpha -1})}^{1/2}\\  &  & \times \,[1-\frac{i}{k+i}(1+{(\frac{i}{k})}^{\alpha -1})+\mathop{\sum }\limits_{n=2}^{\infty }\,{(A{\kappa }_{ik}^{2/5})}^{\frac{n}{2}}{(2({i}^{\alpha -1}+{k}^{\alpha -1}))}^{\frac{n}{20}}\\  &  & \times \,(\frac{(20+n)n}{800})\Gamma (\frac{n}{20})({h}_{n}-\frac{1}{2}{A}_{n}\frac{i}{k+i}(1+{(\frac{i}{k})}^{\alpha -1}))]\end{array}$$

with

21 $$c=\frac{4}{3}\sqrt{2\pi }{\sigma }_{1}^{2}{(\frac{{T}_{1}}{{m}_{1}})}^{\frac{1}{2}}.$$

If the particle size-distribution ${n}_{i}={n}_{i}(i)$ is steep enough the main contribution to the integral in Eq. (20) comes from $i\ll k$. Expanding the integrand in Eq. (20) with respect to $(i/k)\ll 1$ and keeping only the leading terms in the expansion we arrive at (see Methods for more detail):

22 $$\mathop{\sum }\limits_{i=1}^{N}\,{\xi }_{ki}=\{\begin{array}{ll}c{k}^{\frac{\alpha }{2}-\frac{5}{6}}\,{\int }_{1}^{N}\,i\,{n}_{i}\,di & {\rm{if}}\,\alpha \ge 1\\ c{k}^{-\frac{1}{3}}\,{\int }_{1}^{N}\,{i}^{\frac{\alpha +1}{2}}\,{n}_{i}\,di & {\rm{if}}\,0 < \alpha  < 1.\end{array}$$

Here we exclude $\alpha  < 0$, since it may yield for $i\ll k$ a negative sign for the factor in the square brackets of the integrand in Eq. (20). The result of the integration, however, should be positive, as it gives the cooling rate. For steep distributions ${n}_{i}$ one can approximate $N$ in the upper limits of the integrals in Eq. (22) by the infinity,

23 $${\int }_{1}^{N}\,{i}^{p}\,{n}_{i}\,di\simeq {\int }_{1}^{\infty }\,{i}^{p}\,{n}_{i}\,di={\rm{const}},$$

where $p=1$ for $\alpha  > 1$ and $p=(\alpha +1)/2$ for $0 < \alpha  < 1$ (see Eq. (22)), so that the sum in (12) does not (asymptotically, for $N\gg 1$) depend on *N*. Taking into account that the exponents of *k* in the both sides of Eq. (12) must be equal, we finally arrive at:

24 $$\alpha =\{\begin{array}{ll}\frac{5}{9}+\frac{2}{3}\gamma  & {\rm{if}}\,\gamma \ge \frac{2}{3}\\ \gamma +\frac{1}{3} & {\rm{if}}\,-\,\frac{1}{3}\le \gamma \le \frac{2}{3}.\end{array}$$

Surprisingly, this result exactly coincides with the one for the velocity-independent restitution coefficient ${\varepsilon }_{ik}=\varepsilon ={\rm{const}}.$ of ref. ^24^.

In Fig. 3 the theoretical predictions for the temperature distribution are compared with the results of the numerical solution of Eq. (11) with cooling rates of viscoelastic particles, with the cooling rates for the effective quasi-constant restitution coefficient, as well as with the DSMC results (see Methods for the application detail of the DSMC). The results of all three approaches perfectly agree with each other as well as with the theoretical result, Eq. (24). For instance ${T}_{k}\sim {k}^{\mathrm{11/9}}$ for $\gamma =1$ and ${T}_{k}\sim {k}^{\mathrm{17/9}}$ for $\gamma =2$, which corresponds to the case of $\gamma \ge 2/3$ [see Eq. (24)]. Similarly, ${T}_{k}\sim {k}^{\mathrm{5/6}}$ for $\gamma =0.5$, corresponding to −$1/3\le \gamma  < 2/3$, see Fig. 3a. All these temperature distributions are the same as for the case of a velocity-independent restitution coefficient, with the same driving coefficient *γ*.

Interestingly, for the equal distribution of the external energy supply for all species ($\gamma =0$) the energy equipartition does not hold for particles of different sizes: ${T}_{k}\sim {k}^{\mathrm{1/3}}$ which is confirmed both by the scaling prediction and Monte Carlo simulations (Fig. 3b). The reason is that the losses of kinetic energy in collisions of smaller particles is larger than of bigger ones. In order to compensate this effect the input of the external energy should be larger for smaller particles, which can be observed for negative values of *γ*. For the system of viscoelastic particles the equipartition of energy takes place for $\gamma \simeq -\,0.47$ (Fig. 3b), which is slightly different from the value $\gamma \simeq -\,0.33$, predicted by the scaling approach for granular systems with constant restitution coefficient. This resembles the mimicry effect found in the binary mixtures of granular particles^57,58^. For negative values of *γ* with larger absolute values the equipartition breaks again, but the mass-dependence of the granular temperatures becomes inverse: The granular temperature of larger particles becomes smaller^55^. This is indeed observed for the force controlled driving with $\gamma =-\,1$ (Fig. 3b). All these findings are confirmed by the DSMC results.

As we have demonstrated above, the viscoelastic collision model yields the same temperature distribution as the simplified collision model of a constant $\varepsilon$. The same is true for all dissipative mechanisms and may be formulated as a general theorem:

#### **Theorem:**

*Distribution of partial granular temperatures in a driven granular mixture does not depend on the dissipation mechanism of inelastic collisions provided the size distribution of particles is steep enough*.

**Figure 4:**
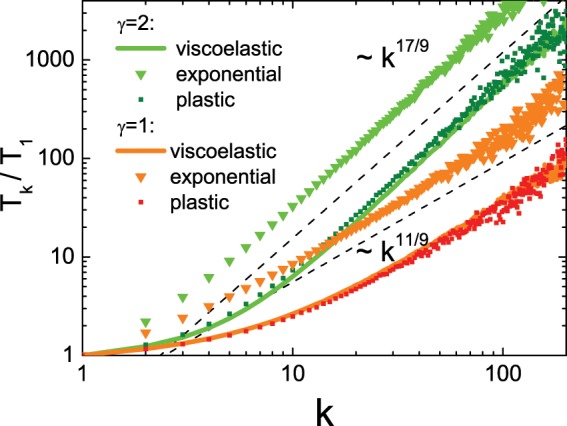
Dependence of the granular temperatures ${T}_{k}$ on the reduced mass of particles $k={m}_{k}/{m}_{1}$ for a heated granular gas for different models of restitution coefficients: the viscoelastic model, elasto-plastic model with ${V}_{{\rm{yield}}}=1$^46^ and the exponential model with $\delta \sqrt{\frac{\rho }{Y}}=1$^47^ obtained in the DSMC simulations. The dashed lines indicate the slopes.

The proof of the theorem and exact formulation of the applicability conditions are given in the section Methods. To illustrate the application of the general theorem we consider the temperature distribution in granular mixtures with other mechanisms of the dissipative collisions – the elasto-plastic model, described by Eq. (5)^46^ and the exponential model, described by Eq. (6)^47^. The results of DSMC simulation for granular mixtures with three different models of the restitution coefficient are shown in Fig. 4.

One can see from the figure that the distribution of granular temperatures demonstrates the same slope for all mechanisms of the dissipative collisions. Moreover, for the case of the first-principle restitution coefficients – for viscoelastic and elasto-plastic dissipative mechanisms, not only the slopes of the distributions, but the distributions themselves coincide. The discrepancy for small $k$ between the temperature distribution of the latter two models and the phenomenological model from ref. ^47^ stems possibly from the unphysically steep decay of $\varepsilon$ with the impact velocity.

## Conclusion

We have studied kinetic properties of size-polydisperse granular mixtures where granular particles suffer pairwise inelastic collisions. These are characterized by the restitution coefficients $\varepsilon$, quantifying the energy losses. We consider two main mechanisms of the collisional dissipation, associated with the viscoelastic and elasto-plastic behavior of the particles material. We used the first-principle expressions for restitution coefficients for these two mechanisms, which implies the dependence of $\varepsilon$ on the impact velocity ${v}_{{\rm{imp}}}$, that is, $\varepsilon =\varepsilon ({v}_{{\rm{imp}}})$. We also considered a phenomenological exponential model for the impact-velocity dependent restitution coefficient $\varepsilon ({v}_{{\rm{imp}}})$ and a simplified model of a constant $\varepsilon$ that does not depend on the impact velocity. We analyzed both cases of force free and driven systems. We derived a system of equations that describe the evolution of granular temperatures of different species in the mixture and the according cooling coefficients ${\xi }_{ij}$ for the case of viscoelastic particles. We have also introduced the notion of the effective impact-velocity independent restitution coefficient $\langle {\varepsilon }_{ij}\rangle$, which significantly simplifies the analysis and demonstrated its adequacy and efficiency. We solved numerically the equations for the granular temperatures ${T}_{k}$, where $k$ specifies the granular species and found the temperature distribution ${T}_{k}$. The theoretical studies have been accompanied by the numerical modeling of the system with the direct simulation Monte Carlo (DSMC). We observed an excellent agreement between the theoretical predictions and the numerical results. Two main conclusions follow from our work: (i) The simplified model of a constant, velocity-independent restitution coefficient fails qualitatively to describe the evolution of a granular mixture and the granular temperature distribution in a homogeneous cooling state (HCS). (ii) Temperature distribution in driven granular mixtures settles to a universal power-law distribution ${T}_{k}\sim {k}^{\alpha }$ with the exponent *α* that does not depend on the dissipation mechanism of inelastic collisions. Moreover, the distribution of temperatures, obtained for the two main dissipation mechanisms – of viscoelastic and plastic energy losses, demonstrate not only coincidence of the slopes but the overall coincidence. This kinetic law is applicable for granular mixtures with a steep size distribution and may be formulated as a general theorem with a precise formulation of the conditions.

The reported results imply serious consequences for the overall kinetic behavior of granular materials – the agitated granular mixtures of very different nature may behave similarly. This result is important for fundamental science, as it helps to understand the kinetic properties of granular mixtures and perform an adequate modelling, as well as for numerous practical applications. As an immediate practical consequence of our findings, is the possibility of experimental modelling (imitation) of natural processes in a lab: One needs to care only about the size distribution of particles and the driving law, but not about particles’ material. Moreover, even if in the course of system evolution, the mechanism of the dissipative collisions changes (e.g. from elasto-plastic to viscoelastic) it will not affect the temperature distribution.

## Methods

### Derivation detail for the cooling coefficients, their sum and effective restitution coefficient

Here we present the derivation detail for obtaining ${\xi }_{ki}$, ${\sum }_{i}\,{\xi }_{ki}$ and $\langle {\varepsilon }_{ik}\rangle$.

#### Cooling coefficients

Due to rather small deviation of the velocity distribution function from the Maxwellian, we assume for this function the Maxwellian form, that is,

25 $${f}_{k}({{\bf{v}}}_{k})={n}_{k}{(\frac{{m}_{k}}{2\pi {T}_{k}})}^{3/2}\,\exp \,(-\frac{{m}_{k}{v}_{k}^{2}}{2{T}_{k}})$$

In order to compute the cooling rate we multiply the Boltzmann equation (Eq. 7) by ${m}_{k}{v}_{k}^{2}\mathrm{/2}$ and integrate it with respect to *v*_*k*_

26 $$\frac{\partial \langle {m}_{k}{v}_{k}^{2}/2\rangle }{\partial t}=\int \,d{{\bf{v}}}_{k}\frac{{m}_{k}{v}_{k}^{2}}{2}\frac{\partial f}{\partial t}=\sum _{i}\,\int \,d{{\bf{v}}}_{k}\frac{{m}_{k}\,{v}_{k}^{2}}{2}{I}_{ki}^{{\rm{coll}}}$$

Using Eq. (8) for the collisional integral we write,

27 $$\begin{array}{rcl}\int \,d{{\bf{v}}}_{k}\frac{{m}_{k}\,{v}_{k}^{2}}{2}{I}_{ki}^{{\rm{coll}}} & = & {\sigma }_{ki}^{2}\,\int \,d{{\bf{v}}}_{k}d{{\bf{v}}}_{i}d{\bf{e}}\Theta (\,-\,{{\bf{v}}}_{ki}\cdot {\bf{e}})\\  &  & \times \,|{{\bf{v}}}_{ki}\cdot {\bf{e}}|\chi f({{\bf{v}}{\boldsymbol{^{\prime\prime} }}}_{k},t)f({{\bf{v}}{\boldsymbol{^{\prime\prime} }}}_{i},t)\frac{{m}_{k}\,{v}_{k}^{2}}{2}\\  &  & -\,{\sigma }_{ki}^{2}\,\int \,d{{\bf{v}}}_{k}d{{\bf{v}}}_{i}d{\bf{e}}\Theta (\,-\,{{\bf{v}}}_{ki}\cdot {\bf{e}})\\  &  & \times \,|{{\bf{v}}}_{ki}\cdot {\bf{e}}|f({{\bf{v}}}_{k},t)f({{\bf{v}}}_{i},t)\frac{{m}_{k}\,{v}_{k}^{2}}{2}.\end{array}$$

Noticing that^10^

28 $$\chi |{{\bf{v}}}_{ki}\cdot {\bf{e}}|d{{\bf{v}}}_{k}d{{\bf{v}}}_{i}=|{{\bf{v}}{\boldsymbol{^{\prime\prime} }}}_{ki}\cdot {\bf{e}}|d{{\bf{v}}{\boldsymbol{^{\prime\prime} }}}_{k}d{{\bf{v}}{\boldsymbol{^{\prime\prime} }}}_{i},$$

we recast the first integral in the r.h.s. of (27) into the form

29 $${\sigma }_{ki}^{2}\,\int \,d{{\bf{v}}{\boldsymbol{^{\prime\prime} }}}_{k}d{{\bf{v}}{\boldsymbol{^{\prime\prime} }}}_{i}d{\bf{e}}\Theta (\,-\,{{\bf{v}}{\boldsymbol{^{\prime\prime} }}}_{ki}\cdot {\bf{e}})|{{\bf{v}}{\boldsymbol{^{\prime\prime} }}}_{ki}\cdot {\bf{e}}|f({{\bf{v}}{\boldsymbol{^{\prime\prime} }}}_{k},t)f({{\bf{v}}{\boldsymbol{^{\prime\prime} }}}_{i},t)\frac{{m}_{k}\,{v}_{k}^{2}}{2}$$

Since the pre-collision velocities ${v^{\prime\prime} }_{k}$ and ${v^{\prime\prime} }_{i}$ are related to ${v}_{k}$ and ${v}_{i}$ in the same way as ${v}_{k}$ and ${v}_{i}$ to post-collision velocities ${v^{\prime} }_{k}$ and ${v^{\prime} }_{i}$, this integral may be rewritten as

30 $${\sigma }_{ki}^{2}\,\int \,d{{\bf{v}}}_{k}d{{\bf{v}}}_{i}d{\bf{e}}\Theta (\,-\,{{\bf{v}}}_{ki}\cdot {\bf{e}})|{{\bf{v}}}_{ki}\cdot {\bf{e}}|f({{\bf{v}}}_{k},t)f({{\bf{v}}}_{i},t)\frac{{m}_{k}\,{v^{\prime} }_{k}^{2}}{2}$$

which finally yields,

31 $$\frac{\partial \langle {m}_{k}{v}_{k}^{2}/2\rangle }{\partial t}=\sum _{i}\,{\sigma }_{ki}^{2}\,\int \,d{{\bf{v}}}_{k}d{{\bf{v}}}_{i}d{\bf{e}}\Theta (\,-\,{{\bf{v}}}_{ki}\cdot {\bf{e}})|{{\bf{v}}}_{ki}\cdot {\bf{e}}|f({{\bf{v}}}_{k},t)f({{\bf{v}}}_{i},t)\Delta {E}_{k}$$

where $\Delta {E}_{k}={m}_{k}{{\bf{v}}{\boldsymbol{^{\prime} }}}_{k}^{2}/2-{m}_{k}{{\bf{v}}}_{k}^{2}/2$ is the difference of energy of a particle of mass ${m}_{k}$ after and before a collision. Let us introduce the center of mass velocity

32 $${\bf{V}}=\frac{{m}_{k}{{\bf{v}}}_{k}+{m}_{i}{{\bf{v}}}_{i}}{{m}_{k}+{m}_{i}}.$$

Using the collision rules, Eq. (2), one can derive $\Delta {E}_{k}$:

33 $$\Delta {E}_{k}=-\,{m}_{{\rm{eff}}}({\varepsilon }_{ki}+1)({{\bf{v}}}_{ki}\cdot {\bf{e}})({\bf{V}}\cdot {\bf{e}})+\frac{1}{2}\frac{{m}_{{\rm{eff}}}^{2}}{{m}_{k}}{({{\bf{v}}}_{ki}\cdot {\bf{e}})}^{2}({\varepsilon }_{ki}^{2}-1)$$

where the restitution coefficient ${\varepsilon }_{ki}$ is given by Eq. (4) with the following elastic constant $\kappa$,

34 $$\kappa =\frac{1}{\sqrt{2}}{(\frac{3}{2})}^{3/2}\frac{Y}{1-{\nu }^{2}}{(\frac{6}{\pi \rho {m}_{1}^{2}})}^{\frac{1}{3}},$$

which is a function of the Young’s modulus $Y$, Poisson ratio $\nu$, monomer mass ${m}_{1}$ and the material density of particles $\rho$^10,36,38^. The dissipative constant $A$ quantifies the viscous properties of the particles’ material^38,41^:

35 $$A=\frac{1}{Y}\frac{(1+\nu )}{(1-\nu )}(\frac{4}{3}{\eta }_{1}(1-\nu +{\nu }^{2})+{\eta }_{2}{(1-2\nu )}^{2})$$

where ${\eta }_{1}$ and ${\eta }_{2}$ are the viscosity coefficients. Let us introduce the variable

36 $${\bf{b}}={\bf{V}}+{{\bf{v}}}_{ki}{m}_{{\rm{eff}}}\frac{{T}_{i}-{T}_{k}}{{m}_{k}{T}_{i}+{m}_{i}{T}_{k}}$$

The change of energy attains the form:

37 $$\begin{array}{rcl}\Delta {E}_{k} & = & {m}_{{\rm{eff}}}({\varepsilon }_{ki}+1)({{\bf{v}}}_{ki}\cdot {\bf{e}})({\bf{b}}\cdot {\bf{e}})\\  &  & +\,{({{\bf{v}}}_{ki}\cdot {\bf{e}})}^{2}\frac{{m}_{{\rm{eff}}}^{2}}{{m}_{k}}(1+{\varepsilon }_{ki})(\frac{1+{\varepsilon }_{ki}}{2}-\frac{{T}_{k}({m}_{k}+{m}_{i})}{{T}_{k}{m}_{i}+{T}_{i}{m}_{k}})\end{array}$$

and the whole integral reads:

38 $$\frac{d{T}_{k}}{dt}=-\,{T}_{k}\,\sum _{i}\,{\xi }_{ki}$$

with

39 $$\begin{array}{rcl}{\xi }_{ki} & = & -\frac{1}{3}{\sigma }_{ki}^{2}{n}_{i}{(\frac{{m}_{k}}{2\pi {T}_{k}})}^{3/2}{(\frac{{m}_{i}}{2\pi {T}_{i}})}^{3/2}\int \,d{{\bf{v}}}_{ki}d{\bf{b}}d{\bf{e}}\Theta (\,-{{\bf{v}}}_{ki}\cdot {\bf{e}})|{{\bf{v}}}_{ki}\cdot {\bf{e}}|\\  &  & \times \,\exp (-\frac{{m}_{k}{m}_{i}}{2({T}_{k}{m}_{i}+{T}_{i}{m}_{k})}{v}_{ki}^{2}-\frac{1}{2}(\frac{{m}_{k}}{{T}_{k}}+\frac{{m}_{i}}{{T}_{i}}){{\bf{b}}}^{2})\frac{\Delta {E}_{k}}{{T}_{k}}.\end{array}$$

Integration that refers to the first term of Δ${E}_{k}$ [see Eq. (37)] yields zero. After the integration over *b* we get:

40 $${\int }_{0}^{\infty }\,d{\bf{b}}\,\exp (-\frac{1}{2}(\frac{{m}_{k}}{{T}_{k}}+\frac{{m}_{i}}{{T}_{i}}){{\bf{b}}}^{2})={(\frac{2\pi {T}_{i}{T}_{k}}{{T}_{k}{m}_{i}+{T}_{i}{m}_{k}})}^{3/2}.$$

And Eq. (39) can be now presented as a sum over the following type of integrals:

41 $$\begin{array}{c}\int \,d{\bf{e}}d{{\bf{v}}}_{ki}\Theta (\,-\,{{\bf{v}}}_{ki}\cdot {\bf{e}})|{{\bf{v}}}_{ki}\cdot {\bf{e}}|{(-{{\bf{v}}}_{ki}\cdot {\bf{e}})}^{2+n/20}\,\exp \,(-R{v}_{ki}^{2})\\ \,=\,4{\pi }^{2}\frac{10}{40+n}{R}^{-3-\frac{n}{20}}\Gamma (3+\frac{n}{20})\end{array}$$

with $R={m}_{k}{m}_{i}/(2({T}_{k}{m}_{i}+{T}_{i}{m}_{k}))$. Collecting all terms together, one gets Eq. (14).

#### Effective restitution coefficient

The derivation of the effective restitution coefficient, Eq. (18), may be performed analogously. Introducing Eq. (4) into Eq. (16), we get

42 $${\varepsilon }_{ki}=1+\frac{1}{\langle {v}_{n}\rangle }\,\mathop{\sum }\limits_{j=1}^{20}\,{h}_{j}{(A{\kappa }_{ki}^{2/5})}^{j/2}{I}_{ki}^{j}$$

with

43 $$\begin{array}{rcl}{I}_{ki}^{j} & = & \int \,d{{\bf{v}}}_{k}d{{\bf{v}}}_{i}d{\bf{e}}\Theta (\,-\,{{\bf{v}}}_{ki}\cdot {\bf{e}}){|{{\bf{v}}}_{ki}\cdot {\bf{e}}|}^{\frac{10+j}{10}}f({{\bf{v}}}_{k},t)f({{\bf{v}}}_{i},t)\\  & = & {(\frac{2({T}_{k}{m}_{i}+{T}_{i}{m}_{k})}{\pi {m}_{k}{m}_{i}})}^{-3/2}\int \,d{\bf{e}}d{{\bf{v}}}_{12}{e}^{-R{v}_{12}^{2}}{|{{\bf{v}}}_{ki}\cdot {\bf{e}}|}^{\frac{10+j}{10}}\\  & = & 2\sqrt{2\pi }{(\frac{{T}_{k}}{{m}_{k}}+\frac{{T}_{i}}{{m}_{i}})}^{1/2}\frac{20}{20+j}\Gamma (2+\frac{j}{20}){2}^{\frac{j}{20}}{(\frac{{T}_{k}}{{m}_{k}}+\frac{{T}_{i}}{{m}_{i}})}^{j/20}.\end{array}$$

#### The sum of cooling rates

To compute the sum ${\sum }_{i}\,{\xi }_{ki}$ in Eq. (11) we analyze the structure of the r.h.s. of Eq. (20) for $i\ll k$, starting from the first term. For $\alpha \ge 1$ the leading term depends on $i$ and $k$ as ${k}^{\alpha \mathrm{/2}-\mathrm{5/6}}i$ and for $\alpha  < 1$ as ${k}^{-1/3}{i}^{(\alpha +1)/2}$. The next terms with $2\le n\le 20$ scale as ${A}^{n/2}{i}^{-n/6}{k}^{(\alpha -1)n/20}$ for $\alpha \ge 1$ and as ${A}^{n/2}{i}^{-(1-\alpha )n/20-1/6}$ for $\alpha  < 1$. For small *A* [which is the condition of the validity of (4)] these terms may be neglected for $n\ge 2$ as compared to the first term, which yields Eq. (22).

### The rigorous prove of the universality of the temperature distribution for all dissipative mechanisms

With mild assumptions we can prove that temperature distribution tends to the scaling form ${k}^{\alpha }$, where *α* does not depend on a particular model of the effective restitution coefficient. Note that effective restitution coefficient exists for every non-negative, continuous and bounded $\varepsilon (v)$ and can be found using Eq. (16), although one can possibly get different values for the effective restitution coefficient and the effective squared restitution coefficient, which we denote respectively as $\langle {\varepsilon }_{ki}\rangle$ and $\langle {\varepsilon }_{ki}^{2}\rangle$ (Here we do not consider the case of very soft particles or nano-particles, which may possess a negative restitution coefficient^59,60^). We present a proof for the Maxwell distribution, but the same techniques can be used in other cases. For simplicity we assume that ${m}_{1}=1$ and ${g}_{2}({\sigma }_{ki})=1$ (the generalization is straightforward), and rewrite (13) as

44 $$\begin{array}{rcl}{\xi }_{ki}(t) & = & \frac{8}{3}\sqrt{2\pi }{n}_{i}{({i}^{1/3}+{k}^{1/3})}^{2}{({T}_{k}/k+{T}_{i}/i)}^{1/2}\\  &  & \times \,(\frac{i}{i+k}[1-\frac{i}{2(i+k)}(1+\langle {\varepsilon }_{ki}\rangle )(1+\frac{{T}_{i}k}{{T}_{k}i})]\\  &  & +\,\frac{i}{i+k}[\langle {\varepsilon }_{ki}\rangle -\frac{i}{2(i+k)}(\langle {\varepsilon }_{ki}\rangle +\langle {\varepsilon }_{ki}^{2}\rangle )(1+\frac{{T}_{i}k}{{T}_{k}i})]).\end{array}$$

First we check the convergence of ${\sum }_{i=1}^{\infty }\,|{\xi }_{ki}|$. Estimating $|{\xi }_{ki}|$ from (44) we find that for $i\geqslant k$

45 $$\mathop{\sum }\limits_{i=k}^{\infty }\,|{\xi }_{ki}|\leqslant {\rm{const}}\cdot \mathop{\sum }\limits_{i=k}^{\infty }\,{n}_{i}{i}^{2/3}\sqrt{1+\frac{{T}_{i}}{i}}\sqrt{1+\frac{{T}_{k}}{k}}(1+\frac{{T}_{i}k}{{T}_{k}i}).$$

Therefore, if ${T}_{i}$ increases with $i$ slower than $i$, we have the convergence if ${\sum }_{i=1}^{\infty }\,{n}_{i}{i}^{2/3} < \infty$. In the opposite case, when ${T}_{i}$ increases with $i$ faster than $i$, the series converges if

46 $$\mathop{\sum }\limits_{i=1}^{\infty }\,{n}_{i}{i}^{-5/6}{T}_{i}^{3/2} < \infty .$$

Let us impose even more restrictions by the condition

47 $$\frac{{T}_{k}\,{\sum }_{i=k}^{\infty }\,|{\xi }_{ki}|}{{k}^{\gamma }}\mathop{\to }\limits^{k\to \infty }0,$$

which holds true, if

48 $$\frac{1}{{k}^{\gamma }}\,\mathop{\sum }\limits_{i=k}^{\infty }\,{n}_{i}{i}^{2/3}({T}_{k}+{T}_{i}\frac{k}{i})\sqrt{1+\frac{{T}_{i}}{i}}\mathop{\to }\limits^{k\to \infty }0.$$

Consider now a partial sum from $i={i}_{0}$ to $k$, which satisfies,

$$\mathop{\sum }\limits_{i={i}_{0}}^{k}\,|{\xi }_{ki}|\leqslant \mathop{\sum }\limits_{i={i}_{0}}^{k}\,{n}_{i}i{k}^{-1/3}\sqrt{1+\frac{{T}_{k}}{k}}$$

and impose the condition

49 $$\forall \epsilon  > 0\,\exists {i}_{0}\,\forall k\geqslant {i}_{0}\,\frac{{T}_{k}\,{\sum }_{i={i}_{0}}^{k}\,|{\xi }_{ki}|}{{k}^{\gamma }} < \epsilon ,$$

which is true if ${\sum }_{i=1}^{\infty }\,{n}_{i}i < \infty$, and ${T}_{k}=O({k}^{\alpha })$ for $\alpha$ from (24). Combining conditions (47) and (49) we observe that

50 $$\forall \epsilon  > 0\,\forall k\geqslant {i}_{0}\,\frac{{T}_{k}\,{\sum }_{i={i}_{0}}^{\infty }\,|{\xi }_{ik}|}{{k}^{\gamma }} < \epsilon ,$$

which essentially means that the full series may be replaced by its first ${i}_{0}$ terms with any desired accuracy $\epsilon$ (${i}_{0}$ certainly depends on $\epsilon$). Hence for $k\gg {i}_{0}$ we have the same asymptotics for ${T}_{k}$, obtained for the incomplete sum, as the one obtained for the whole series. These differ only by the factor $1+\epsilon$, converging to $1$ as ${i}_{0}$ and $k$ increase (hereinafter it is implied that $\epsilon$ may be taken arbitrarily small).

Now we illustrate that for $k\gg {i}_{0}$ the terms of ${\xi }_{ki}$ (44) do converge. We have

$$\begin{array}{l}\begin{array}{rcl}{({i}^{1/3}+{k}^{1/3})}^{2} & = & (1+\epsilon ){k}^{2/3}\\ \sqrt{\frac{{T}_{i}}{i}+\frac{{T}_{k}}{k}} & = & \{\begin{array}{ll}(1+\epsilon )\sqrt{\frac{{T}_{i}}{i}}, & {\rm{if}}\,{T}_{k}/k\to 0\\ (1+\epsilon )\sqrt{{\rm{const}}+\frac{{T}_{k}}{k}}, & {\rm{otherwise}}\end{array}\\ \frac{i}{i+k} & = & (1+\epsilon )\frac{1}{k}\end{array}\\ \frac{i}{i+k}\cdot \frac{{T}_{i}k}{{T}_{k}i}\to 0\,{\rm{if}}\,{T}_{k}\to \infty \end{array}.$$

The last condition means that the terms with the negative sign in (44) disappear. Then we are left with

51 $${\xi }_{ki}(t)={\rm{const}}\cdot (1+\epsilon ){k}^{-1/3}\sqrt{\frac{{T}_{i}}{i}+\frac{{T}_{k}}{k}}(1+\langle {\varepsilon }_{ki}\rangle ),\,k\gg 1.$$

If $\langle {\varepsilon }_{ki}\rangle$ is continuous and has a limit for $k\to \infty$, then $1+\langle {\varepsilon }_{ki}\rangle$ is also a a value (that depends only on $i$) between 1 and 2 up to the factor $(1+\epsilon )$. Solving then the stationary Eq. (12) for ${T}_{k}$,

52 $${T}_{k}\,\sum _{i}\,{\xi }_{ki}={\Gamma }_{1}{k}^{\gamma }$$

with ${\xi }_{ki}$ from (51) leads to

$${T}_{k}={\rm{const}}\cdot (1+\epsilon ){k}^{\alpha }$$

with *α* from (24), which proves the asymptotics.

Note that the special case of $\alpha =1$ is to be treated with an additional care. In this case the limit of $\langle {\varepsilon }_{ik}\rangle$ for $k\to \infty$ may depend on the limit of ${T}_{k}/{k}^{\alpha }$; Eq. (52) for ${T}_{k}$, with ${\xi }_{ki}$ from (51), turns into an equation for an implicit function $f(x,\epsilon )$ with $x={T}_{k}/{k}^{\alpha }$. In this particular case one also needs to check that ${\xi }_{ki}$, given by Eq. (51) is a monotonous function of $x={T}_{k}/{k}^{\alpha }$. Otherwise, multiple solutions of Eq. (52) for $\mathop{\mathrm{lim}}\limits_{k\to \infty }\,\frac{{T}_{k}}{{k}^{\alpha }}$ may exist. In all the addressed above cases, the quantity $\langle {\varepsilon }_{ik}\rangle$ decreases slower than $1-c|\overrightarrow{v}|$; hence the monotony is guaranteed, so that $\mathop{\mathrm{lim}}\limits_{k\to \infty }\,\frac{{T}_{k}}{{k}^{\alpha }}={\rm{const}} > 0$ even for $\alpha =1$.

Substituting ${T}_{k}\sim {k}^{\alpha }$ into (46) and (48) we see that the following conditions are sufficient for the convergence for the obtained asymptotics:

1. $${n}_{i}=O(1/{i}^{{\rm{\max }}(2,\gamma +1)+\delta }),\,{\rm{with}}\,\delta  > 0$$
2. $$\gamma  > -\,1/3$$

Surprisingly, there are no conditions imposed on the effective restitution coefficients $\langle {\varepsilon }_{ik}\rangle$, except for the natural ones – continuity and existence of some limit for high speeds, which are obviously satisfied due to physical reasons. It should be also noted however that the power-law asymptotics is valid only for large $k$ and is not expected for $k\sim 1$. In our simulations we used ${n}_{k}\sim {k}^{-3}$, which means that the monomers dominate in the system and the convergence to the asymptotics is already visible for rather small $k$.

### Direct simulation monte carlo

To study the granular mixtures we implement the method of Direct Simulation Monte Carlo, which is widely used in investigations of granular systems, especially for granular gases, where it provides very accurate results^53,61^. It is based on the solution of the Boltzmann equation for space uniform systems by stochastic methods^53,62^. Generally, we follow the standard procedure^62^, which has been adopted accordingly for the granular mixture. Here we give the simulation detail and the main ideas of the approach. We used 2.7 × 10^8^ monomers, so that for the size distribution ${n}_{k}\sim 1/{k}^{3}$ addressed here, one has ${N}_{k}=\lfloor {N}_{1}/{k}^{3}\rfloor =\lfloor 2.7\cdot {10}^{8}/{k}^{3}\rfloor$ *k*-mers. The maximum size in our simulations was ${k}_{{\rm{\max }}}=300$. We use the following parameters: the mass of monomer ${m}_{1}=0.01$, its diameter, ${\sigma }_{1}=1$, the number density of monomers ${n}_{1}=0.1$ and the initial temperature ${T}_{0}=1$. These parameters correspond to a dilute granular mixture with the packing fraction of monomers $\phi =(\pi /6){n}_{1}{\sigma }_{1}^{3}=0.0524$, which guarantees the accuracy of the Boltzmann equation^10^. Note that any sufficiently small packing fraction leads to the same distribution, since scaling number density leads to the same changes in the equations and simulations as changing the time scale by the same amount.

A stochastic thermostat has been implemented through the random change of velocities of all particles every *N* *h* collisions, where $N$ is the total number of particles and $h$ is a parameter, which should be much less than one so that the thermostat affects any particular particle several times between its collisions. Here we choose $h=0.1$.

The velocity components of each particle changes each *N* *h* collisions:

$$\begin{array}{lll}{v}_{x} & := & {v}_{x}+{r}_{1}\sqrt{{\Gamma }_{i}\Delta t/{m}_{i}},\\ {v}_{y} & := & {v}_{y}+{r}_{2}\sqrt{{\Gamma }_{i}\Delta t/{m}_{i}},\\ {v}_{z} & := & {v}_{z}+{r}_{3}\sqrt{{\Gamma }_{i}\Delta t/{m}_{i}},\end{array}$$

where Δ*t* is the time passed after previous velocity change due to the thermostat, and ${r}_{1,2,3}$ are random variables from a normal distribution with zero mean and unit standard deviation, that is, ${r}_{1,2,3}\in N(0,1)$.

We consider only binary collisions of the particles and neglect possible triple and higher order collisions. Colliding particles are chosen in two steps:

1. We choose the sizes of the colliding particles.
2. We choose the speeds of the colliding particles.

Let us discuss each step in more detail.

1. We can estimate the number of candidate pairs $(ij)$ to collide during the small time interval Δ${t}_{ij}$ as
  53 $$\begin{array}{rcl}{N}_{p}^{ij} & = & \frac{\pi {(\frac{{\sigma }_{i}+{\sigma }_{j}}{2})}^{2}{N}_{i}{N}_{j}(|{v}_{i}{|}_{{\rm{\max }}}+|{v}_{j}{|}_{{\rm{\max }}})}{V}\Delta {t}_{ij}\\  & = & \pi \frac{{n}_{1}}{{N}_{1}}{N}_{i}{N}_{j}{\sigma }_{1}^{2}{(\frac{{i}^{1/3}+{j}^{1/3}}{2})}^{2}(|{v}_{i}{|}_{{\rm{\max }}}+|{v}_{j}{|}_{{\rm{\max }}})\Delta {t}_{ij},\end{array}$$
  where $(1/4)\pi {({\sigma }_{i}+{\sigma }_{j})}^{2}(|{v}_{i}{|}_{{\rm{\max }}}+|{v}_{j}{|}_{{\rm{\max }}})\Delta {t}_{ij}$ gives the volume of the collision cylinder and we use the definition of the number density, ${n}_{i}={N}_{i}/V$ (*V* is the system volume). Let us introduce the matrix ${\Lambda }_{ij}$ of collision rates. They are equal to the inverse of the average time of one collision between particles of sizes $i$ and $j$, that is, ${\Lambda }_{ij}={N}_{p}^{ij}\Delta {t}_{ij}^{-1}$. Then we get from (53):
  $${\Lambda }_{ij}=\pi \frac{{n}_{1}}{{N}_{1}}{N}_{i}{N}_{j}{\sigma }_{1}^{2}{(\frac{{i}^{1/3}+{j}^{1/3}}{2})}^{2}(|{v}_{i}{|}_{{\rm{\max }}}+|{v}_{j}{|}_{{\rm{\max }}}).$$
  The time between collisions and the sizes of colliding particles are chosen based on matrix $\parallel {\Lambda }_{ij}\parallel$. Namely, the time between collisions is taken from the exponential distribution
  $$\Delta t=-\,\mathrm{ln}({\rm{rand}}(0,1])/\mathop{\sum }\limits_{i,j=1}^{{m}_{{\rm{\max }}}}\,{\Lambda }_{ij}$$
  and the sizes $i$ and $j$ of the colliding particles are chosen with probability
  $${p}_{ij}=\frac{{\Lambda }_{ij}}{{\sum }_{i,j=1}^{{m}_{{\rm{\max }}}}\,{\Lambda }_{ij}}.$$
  Note that matrix $\parallel {\Lambda }_{ij}\parallel$ has rank 6 and can be rapidly recalculated using the low-rank technique (see e.g.^63^).
2. When the sizes $i$ and $j$ of the colliding particles are known, we can estimate the maximum value of the relative velocity of colliding particles according to

$$|{v}_{ij}{|}_{{\rm{\max }}}\leqslant |{v}_{i}{|}_{{\rm{\max }}}+|{v}_{j}{|}_{{\rm{\max }}}.$$

Then we use the standard technique to pick random particles and accept the collision if

$$|{\bf{e}}({{\bf{v}}}_{i}-{{\bf{v}}}_{j})| > {\rm{rand}}[0,1)(|{v}_{i}{|}_{{\rm{\max }}}+|{v}_{j}{|}_{{\rm{\max }}}),$$

where **e** is a random unit vector. The values of $|{{\bf{v}}}_{i}{|}_{{\rm{\max }}}$ can be updated in $O(\log \,{N}_{i})$ steps by keeping all velocities in a binary heap structure for each size.

To reduce the statistical noise of the simulation data for temperatures we apply the running averaging as follows. Let at time ${t}_{{\rm{conv}}}$ the temperature of monomers ceases to change monotonously. This indicates that the system has achieved its steady state and the stochastic noise becomes the primary source of errors. We then calculate the temperatures every $N$ collisions in the time interval $({t}_{{\rm{conv}}},2{t}_{{\rm{conv}}})$ and take an average^64,65^.

## References

[1] Herrmann HJ, Hovi J-P, Luding S (1998). Physics of Dry Granular Media..

[2] Jaeger H, Nagel S, Behringer R (1996). Granular solids, liquids, and gases. Rev. Mod. Phys..

[3] Hinrichsen H, Wolf DE (2004). The Physics of Granular Media..

[4] Duran J (2000). Sands, Powders and Grains.

[5] Apai, D. & Lauretta, D. S. *Protoplanetary Dust. Astrophysical and Cosmochemical Perspectives*. (Cambridge University Press, 2010).

[6] Bridges FG, Hatzes A, Lin DNC (1984). Structure, stability and evolution of Saturn’s rings. Nature.

[7] Brilliantov N (2015). Size distribution of particles in Saturn’s rings from aggregation and fragmentation. Proc. Natl. Acad. Sci. USA.

[8] Heiken, G. H., Vanniman, D. T. & French, B. M. *Lunar Sourcebook*. (Cambridge University Press, 1991).

[9] Almeida MP, Parteli EJR, Andrade JS, Herrmann HJ (2008). Giant saltation on Mars. Proc. Natl. Acad. Sci. USA.

[10] Brilliantov NV, Pöschel T (2004). Kinetic theory of Granular Gases..

[11] Tai Y-C, Hutter K, Gray JMNT (2001). Geomorphological Fluid Mechanics. Lecture Notes in Physics.

[12] Wiggs GFS (2001). Desert dune processes and dynamics. Progress in Physical Geography.

[13] Brey JJ, Ruiz-Montero MJ, Garcia-Rojo R, Dufty JW (1999). Brownian motion in a granular gas. Phys. Rev. E.

[14] Dufty JW, Brey JJ (2005). Brownian motion in a granular fluid. New J. of Phys..

[15] Garzo V, Dufty JW (1999). Homogeneous cooling state for a granular mixture. Phys. Rev. E.

[16] Dahl SR, Hrenya CM, Garzo V, Dufty JW (2002). Kinetic temperatures for a granular mixture. Phys. Rev. E.

[17] Wildman RD, Parker DJ (2002). Coexistence of Two Granular Temperatures in Binary Vibrofluidized Beds. Phys. Rev. Lett.

[18] Feitosa K, Menon N (2002). Breakdown of Energy Equipartition in a 2D Binary Vibrated Granular Gas. Phys. Rev. Lett.

[19] Schmidt, J., Ohtsuki, K., Rappaport, N., Salo, H. & Spahn, F. *Dynamics of Saturn’s Dense Rings*. In: Dougherty, M. K., Esposito, L. W. & Krimigis, S. M. (Eds.) *Saturn from Cassini-Huygens* (Springer) **413** (2009).

[20] Ohtsuki K (1999). Evolution of Particle Velocity Dispersion in a Circumplanetary Disk Due to Inelastic Collisions and Gravitational Interactions. Icarus.

[21] Ohtsuki K (2006). Rotation rate and velocity dispersion of planetary ring particles with size distribution II. Numerical simulation for gravitating particles. Icarus.

[22] Salo H (1992). Numerical simulations of dense collisional systems: II. Extended distribution of particle sizes. Icarus.

[23] Spahn F, Albers N, Sremčević M, Thornton C (2004). Kinetic description of coagulation and fragmentation in dilute granular particle ensembles. Europhys. Lett..

[24] Bodrova A, Levchenko D, Brilliantov NV (2014). Universality of temperature distribution in granular gas mixtures with a steep particle size distribution. Europhys. Lett..

[25] Haff P (1983). Grain flow as a fluid-mechanical phenomenon. J. Fluid Mech..

[26] Goldhirsch I, Zanetti G (1993). Clustering instability in dissipative gases. Phys. Rev. Lett..

[27] Das S, Puri S (2003). Pattern formation in the inhomogeneous cooling state of granular fluids. Europhys. Lett..

[28] Das S, Puri S (2003). Kinetics of inhomogeneous cooling in granular fluids. Phys. Rev. E.

[29] Nakanishi H (2003). Velocity distribution of inelastic granular gas in a homogeneous cooling state. Phys. Rev. E.

[30] van Noije T, Ernst M, Brito R, Orza J (1997). Mesoscopic Theory of Granular Fluids. Phys. Rev. Lett..

[31] van Noije T, Ernst M, Brito R (1998). Spatial correlations in compressible granular flows. Phys. Rev. E.

[32] Ahmad S, Puri S (2006). Velocity distributions in a freely evolving granular gas. Europhys. Lett..

[33] Ahmad S, Puri S (2007). Velocity distributions and aging in a cooling granular gas. Phys. Rev. E.

[34] Goldsmit W (2004). The Theory and Physical Behavior of Colliding Solids.

[35] Kuwabara G, Kono KJ (1987). Restitution Coefficient in a Collision between TwoSpheres. Appl. Phys. Part 1.

[36] Ramirez R, Pöschel T, Brilliantov N, Schwager T (1999). Coefficient of restitution of colliding viscoelastic spheres. Phys. Rev. E.

[37] Tanaka T, Ishida T, Tsuji Y (1991). Trans. Direct Numerical Simulation of Granular Plug Flow in a Horizontal Pipe: the Case of Cohesionless Particles. Jap. Soc. Mech. Eng..

[38] Brilliantov N, Spahn F, Hertzsch J, Pöschel T (1996). Model for collisions in granular gases. Phys. Rev. E.

[39] Morgado W, Oppenheim I (1997). Energy dissipation for quasielastic granular particle collisions. Phys. Rev. E.

[40] Schwager T, Pöschel T (1998). Coefficient of normal restitution of viscous particles and cooling rate of granular gases. Phys. Rev. E.

[41] Goldobin DS, Susloparov EA, Pimenova AV, Brilliantov NV (2015). Collision of viscoelastic bodies: Rigorous derivation of dissipative force. Eur. Phys. J. E.

[42] Schwager T, Pöschel T (2008). Coefficient of restitution for viscoelastic spheres: The effect of delayed recovery. Phys. Rev. E.

[43] Falcon E, Laroche C, Fauve S, Coste C (1998). Behavior of one inelastic ball bouncing repeatedly off the ground. Eur. Phys. J. B.

[44] Labous L, Rosato AD, Dave RN (1997). Measurements of collisional properties of spheres using high-speed video analysis. Phys. Rev. E.

[45] McNamara S, Falcon E (2008). Simulations of dense granular gases without gravity with impact-velocity-dependent restitution coefficient. Powder Technology.

[46] Thornton C, Ning Z (1998). A theoretical model for the stick/bounce behaviour of adhesive, elastic-plastic spheres. Powder Technology.

[47] Lun CKK, Savage SB (1986). The Effects of an Impact Velocity Dependent Coefficient of Restitution on Stresses Developed by Sheared Granular Materials. Acta Mechanica.

[48] Brilliantov NV, Pöschel T (2000). Velocity distribution in granular gases of viscoelastic particles. Phys. Rev. E.

[49] Dubey AK, Bodrova A, Puri S, Brilliantov N (2013). Velocity distribution function and effective restitution coefficient for a granular gas of viscoelastic particles. Phys. Rev. E.

[50] Brilliantov NV, Poeschel T (2000). Self-diffusion in granular gases. Phys. Rev. E.

[51] Brilliantov NV, Saluena C, Schwager T, Poeschel T (2004). Transient structures in a granular gas. Phys. Rev. Lett..

[52] Williams DRM, MacKintosh FC (1996). Driven granular media in one dimension: Correlations and equation of state. Phys. Rev. E.

[53] Montanero JM, Santos A (2000). Computer simulation of uniformly heated granular fluids. Gran. Mat..

[54] van Noije T, Ernst M (1998). Velocity distributions in homogeneous granular fluids: the free and the heated case. Gran. Mat..

[55] Uecker H, Kranz WT, Aspelmeier T, Zippelius A (2009). Partitioning of energy in highly polydisperse granular gases. Phys. Rev. E.

[56] Cafiero R, Luding S, Herrmann HJ (2000). Two-Dimensional Granular Gas of Inelastic Spheres with Multiplicative Driving. Phys. Rev. Lett..

[57] Lasanta, A., Vega Reyes, F., Garzo, V. & Santos, A. Intruders in disguise: Mimicry effect in granular gases, https://arxiv.org/abs/1903.10807.

[58] Megias, A. & Santos, A. Driven and undriven states of multicomponent granular gases of inelastic and rough hard disks or spheres, https://arxiv.org/abs/1901.11307.

[59] Saitoh K, Bodrova A, Hayakawa H, Brilliantov NV (2010). Negative Normal Restitution Coefficient Found in Simulation of Nanocluster Collisions. Phys. Rev. Lett..

[60] Muller P, Krengel D, Poschel T (2012). Negative coefficient of normal restitution. Phys. Rev. E.

[61] Brilliantov N, Poeschel T, Formella A (2018). Increasing temperature of cooling granular gases. Nature Communication.

[62] Poschel T, Schwager T (2005). Computational Granular Dynamics..

[63] Horn, R. A. & Roger, C. R. *Matrix analysis*. Second edition. (Cambridge University Press, 2013).

[64] Grasselli Y, Bossis G, Goutallier G (2009). Velocity-dependent restitution coefficient and granular cooling in microgravity. Europhys. Lett..

[65] Bodrova AS, Dubey AK, Puri S, Brilliantov NV (2012). Intermediate Regimes in Granular Brownian Motion: Superdiffusion and Subdiffusion. Phys. Rev. Lett..

